# Methotrexate-Induced Leukocytoclastic Vasculitis

**DOI:** 10.7759/cureus.16519

**Published:** 2021-07-20

**Authors:** Pooja Dewan, Sunil Gomber, Maharshi Trivedi, Preeti Diwaker, Ujjwal Madan

**Affiliations:** 1 Pediatrics, University College of Medical Sciences, Delhi, IND; 2 Pediatrics/Oncology, University College of Medical Sciences, Delhi, IND; 3 Pathology, University College of Medical Sciences, Delhi, IND

**Keywords:** cancer, chemotherapy, child, rash, vasculitis

## Abstract

Erythematous tender cutaneous lesions developed in a 10-year-old child of acute leukemia receiving oral methotrexate and 6-mercaptopurine during maintenance phase of chemotherapy. She was also found to have coagulopathy and transaminitis. Differential clinical diagnosis included infectious processes, pyoderma gangrenosum, connective tissue disorders like rheumatoid neutrophilic dermatitis, and drug-induced side effects. Oral methotrexate was withheld following which the lesions subsided. Skin biopsy revealed a diagnosis of leukocytoclastic vasculitis. Cutaneous vasculitis is a rare side effect of methotrexate and its possibility should be considered in any patient who develops skin lesions while being receiving chemotherapy.

## Introduction

Methotrexate is an anti-metabolite drug that had a variety of clinical applications, including anti-inflammatory, immunomodulatory, and anti-neoplastic effects [1]. Methotrexate and its polyglutamate metabolites bind to the enzyme dihydrofolate reductase and inhibit nucleotide synthesis; decreased cell division and multiplication in turn are responsible for the therapeutic effects. In children, methotrexate is prescribed for treatment of lymphoreticular malignancies like acute lymphoblastic leukemia (ALL) as well as various rheumatic diseases like juvenile idiopathic arthritis (JIA), juvenile dermatomyositis (JDM), localized scleroderma, and psoriasis. Methotrexate is also used to treat vasculitis associated with rheumatic illnesses [2-4].

However, methotrexate is a toxic drug and its use is fraught with adverse effects like hepatotoxicity, nephrotoxicity, neurotoxicity, and bone marrow suppression [5]. Uncommonly, methotrexate has been reported to cause cutaneous side effects, including photosensitivity, alopecia, pruritus, mucositis, alterations in skin pigmentation, and vasculitis [6]. There are only few reports of histologically proven methotrexate-associated vasculitis [7-11], especially in children with hematological malignancies [9]. Herein, we present a case of a child with acute biphenotypic leukemia who developed histologically proven leukocytoclastic vasculitis (LCV) while receiving low-dose oral methotrexate therapy.

## Case presentation

A 10-year-old girl was diagnosed with acute biphenotypic leukemia according to the World Health Organization criteria [12]. The flow cytometric analysis of cluster of differentiation (CD) markers in the bone marrow aspirate revealed the presence of lymphoblasts that co-expressed key markers of B-cell lymphoid and myeloid lineage, i.e., positive for CD19, CD79a, CD10, myeloperoxidase (MPO), and terminal deoxynucleotidyl transferase (TdT) but negative for CD3, CD5, and CD7 [12]. She was started on treatment with modified Multicenter protocol-841 (MCP-841) [13]. She completed the initial 20 weeks of treatment wherein she received intravenous (IV) vincristine, IV daunomycin, IV cytosine arabinoside, IV L-asparaginase, IV cyclophosphamide, intrathecal methotrexate, oral prednisolone, and oral mercaptopurine at scheduled dates as per protocol. At the end of the consolidation phase (end of 20th week), the child was in clinical as well as morphological remission proven on bone marrow evaluation.

**Figure 1:**
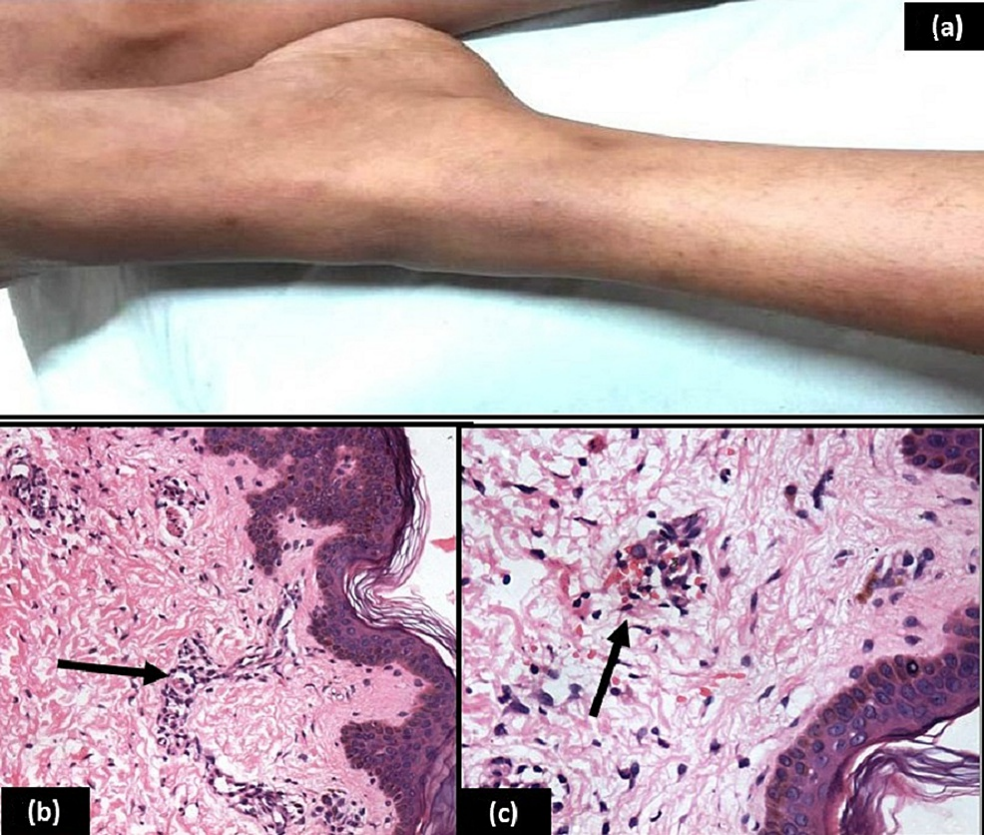
(a) Erythematous skin lesions on both legs. (b) H& E 200x: Section shows upper dermal perivascular inflammatory infiltrate comprised of neutrophils and lymphocytes at places invading into the vessel wall (arrow). (c): H& E 400x: Section shows upper dermal perivascular inflammatory infiltrate along with extravasation of red blood cells (arrow).

However, she developed itchy reddish-blue macules and papules over both upper and lower limbs during the maintenance phase of chemotherapy (week 24 of treatment) wherein she was receiving oral 6-mercaptopurine (60 mg/m^2^/day), and oral methotrexate (15 mg/m^2^ weekly) (Figure 1a). There was no associated fever, bleeding, jaundice, or excessive paleness. On examination, multiple, tender, erythematous, palpable lesions, about 3×4 cm in size were present over both legs and arms of the child. The rest of the systemic examination was unremarkable. Laboratory investigations revealed hemoglobin 8.2 g/dL, total leucocyte count 1900/mm^3^, platelet count 128,000/mm^3^; prothrombin time (PT) 25.2s, activated partial thromboplastin time (aPTT) > 60s, international normalised ratio (INR) 2.27, total bilirubin 0.4 mg/dL, serum glutamate pyruvate transaminase (SGPT) 403 IU/dL, serum alkaline phosphatase (ALP) 138 IU/dL, and serum albumin 3.4 g/dL. Hepatitis B surface antigen and hepatitis C antibodies were non-reactive. Coomb’s antibodies, rheumatoid factor, and antinuclear antibodies were also not detected. Ultrasonography of the abdomen revealed mild hepatomegaly with normal echotexture. In the absence of any other identifiable cause, a possibility of methotrexate-induced hepatotoxicity and vasculitis was considered, and chemotherapy was temporarily withheld. Skin biopsy was performed which revealed mild perivascular inflammatory infiltrate comprised of neutrophils, lymphocytes at places invading into the vessel wall along with focal red blood cell extravasation suggestive of LCV (Figures 1b, 1c). Two weeks following cessation of chemotherapy, PT, aPTT, and INR were 14.0s, 29.1s, and 1.27, respectively; SGPT was 315 IU/dL and ALP was 133 IU/dL. The skin lesions had also subsided with oral anti-histaminic drugs. Chemotherapy was resumed after two weeks. The child, however, again developed skin lesions. We decided to continue the chemotherapy as the lesions were much lesser and were controlled with symptomatic therapy using oral hydroxyzine hydrochloride and topic application of calamine.

## Discussion

Leukocytoclastic vasculitis, also known as hypersensitivity vasculitis (or angiitis), is a small vessel vasculitis of dermal capillaries and venules, which presents as palpable purpura usually on the lower limbs. It can also manifest as urticaria, hemorrhagic vesicles, ulcers, nodules, livedo, infarcts, or digital gangrene. Histologically, LCV is characterized by leukocytoclasis, which refers to vascular damage caused by nuclear debris from infiltrating neutrophils.

Approximately half of the cases of LCV are idiopathic; the causative factors in the remainder include infections, food or food additives, collagen vascular diseases, malignancies, and drugs. LCV has been reported in patients with leukemia, lymphoma, sarcoma, multiple myeloma, hairy cell leukemia, and solid organ tumors [14]. The pathogenesis of LCV may be chemotherapy-related or due to the neoplasm itself [15], or paraneoplastic effects [16]. Drug-induced LCV has been reported to be due to antibiotics (like beta-lactams, erythromycin, clindamycin, vancomycin, and sulfonamides), non-steroidal anti-inflammatory drugs, diuretics, and anticonvulsants like phenytoin and valproic acid. Hydroxyurea, vincristine, cytosine arabinoside, busulphan, all-trans retinoic acid, and granulocyte colony-stimulating factor have also been implicated for LCV [17]. Methotrexate has only uncommonly been reported to cause LCV, especially with its use in low doses. Most of the cases of methotrexate-associated LCV have been associated with protracted infusion schedules or the use of high or intermediate doses [7-10].

In our child, the LCV was probably because of oral methotrexate therapy, as on the Naranjo causality scale for adverse drug reactions the score turned out to be 6 as shown in Table 1 [18]. LCV does not appear to be linked with the lymphoreticular malignancy in our child as her bone marrow examination suggested hematological remission. The clinical and histological findings, the temporal relationship between methotrexate intake and the onset of vasculitis, and the results of withdrawal and re-challenge tests suggest a causal relationship in our case [11]. Paradoxically, methotrexate has also been used for the treatment of LCV in patients with connective tissue disorders [2-4]. Though LCV is not life-threatening toxicity and most cases improve spontaneously, our child also developed transaminitis and deranged coagulation profile which were also attributed to methotrexate, and therefore her chemotherapy was withheld temporarily.

**Table 1:** Naranjo adverse drug reaction probability scale as applied to our case The adverse drug reaction is assigned to a probability category from the total score as follows: definite if the overall score is 9 or greater, probable for a score of 5-8, possible for 1-4, and doubtful if the score is 0.

To assess the adverse drug reaction, please answer the following questionnaire and give the pertinent score	Yes	No	Do not know	Score in our case
Are there previous conclusive reports on this reaction?	+1	0	0	+1
Did the adverse event occur after the suspected drug was administered?	+2	-1	0	+2
Did the adverse reaction improve when the drug was discontinued or a specific antagonist was administered?	+1	0	0	+1
Did the adverse reaction reappear when the drug was readministered?	+2	-1	0	+2
Are there alternative causes (other than the drug) that could have on their own caused the reaction?	-1	+2	0	0
Did the reaction reappear when a placebo was given?	-1	+1	0	-1
Was the blood detected in the blood (or other fluids) in concentrations known to be toxic?	+1	0	0	0
Was the reaction more severe when the dose was increased or less severe when the dose was decreased?	+1	0	0	0
Did the patient have a similar reaction to the same or similar drugs in any previous exposure?	+1	0	0	0
Was the adverse event confirmed by any objective evidence?	+1	0	0	+1

Most of these effects are observed when methotrexate is administered in high dose, although our child was receiving methotrexate in a weekly dose of 15 mg/m^2^. Immediate type of hypersensitivity has been reported as pathogenesis for LCV caused by methotrexate [7,11].

## Conclusions

Methotrexate-induced leukocytoclastic vasculitis should be considered as a possible etiology in patients with lymphoreticular malignancies who develop rash while receiving chemotherapy. It is usually a self-limiting condition and may rarely warrant discontinuation of therapy only transiently.
